# Testosterone therapy induces molecular programming augmenting physiological adaptations to resistance exercise in older men

**DOI:** 10.1002/jcsm.12472

**Published:** 2019-09-30

**Authors:** Nima Gharahdaghi, Supreeth Rudrappa, Matthew S. Brook, Iskandar Idris, Hannah Crossland, Claire Hamrock, Muhammad Hariz Abdul Aziz, Fawzi Kadi, Janelle Tarum, Paul L. Greenhaff, Dumitru Constantin‐Teodosiu, Jessica Cegielski, Bethan E. Phillips, Daniel J. Wilkinson, Nathaniel J. Szewczyk, Kenneth Smith, Philip J. Atherton

**Affiliations:** ^1^ MRC‐ARUK Centre for Musculoskeletal Ageing Research and Nottingham NIHR BRC, School of Medicine University of Nottingham, Derby UK; ^2^ Institute of Food and Health University College Dublin, Belfield Dublin Ireland; ^3^ Division of Sports Sciences, School of Health and Medical Sciences Örebro University Örebro Sweden; ^4^ MRC‐ARUK Centre for Musculoskeletal Ageing Research, School of Life Sciences University of Nottingham, Nottingham Nottingham UK

**Keywords:** Skeletal muscle, Protein turnover, Testosterone, Resistance exercise, Ageing

## Abstract

**Background:**

The andropause is associated with declines in serum testosterone (T), loss of muscle mass (sarcopenia), and frailty. Two major interventions purported to offset sarcopenia are anabolic steroid therapies and resistance exercise training (RET). Nonetheless, the efficacy and physiological and molecular impacts of T therapy adjuvant to short‐term RET remain poorly defined.

**Methods:**

Eighteen non‐hypogonadal healthy older men, 65–75 years, were assigned in a random double‐blinded fashion to receive, biweekly, either placebo (P, saline, *n* = 9) or T (Sustanon 250 mg, *n* = 9) injections over 6 week whole‐body RET (three sets of 8–10 repetitions at 80% one‐repetition maximum). Subjects underwent dual‐energy X‐ray absorptiometry, ultrasound of vastus lateralis (VL) muscle architecture, and knee extensor isometric muscle force tests; VL muscle biopsies were taken to quantify myogenic/anabolic gene expression, anabolic signalling, muscle protein synthesis (D_2_O), and breakdown (extrapolated).

**Results:**

Testosterone adjuvant to RET augmented total fat‐free mass (*P=*0.007), legs fat‐free mass (*P=*0.02), and appendicular fat‐free mass (*P=*0.001) gains while decreasing total fat mass (*P=*0.02). Augmentations in VL muscle thickness, fascicle length, and quadriceps cross‐section area with RET occured to a greater extent in T (*P* < 0.05). Sum strength (*P=*0.0009) and maximal voluntary contract (e.g. knee extension at 70°) (*P=*0.002) increased significantly more in the T group. Mechanistically, both muscle protein synthesis rates (T: 2.13 ± 0.21%·day^−1^ vs. P: 1.34 ± 0.13%·day^−1^, *P=*0.0009) and absolute breakdown rates (T: 140.2 ± 15.8 g·day^−1^ vs. P: 90.2 ± 11.7 g·day^−1^, *P=*0.02) were elevated with T therapy, which led to higher net turnover and protein accretion in the T group (T: 8.3 ± 1.4 g·day^−1^ vs. P: 1.9 ± 1.2 g·day^−1^, *P=*0.004). Increases in ribosomal biogenesis (RNA:DNA ratio); mRNA expression relating to T metabolism (androgen receptor: 1.4‐fold; *Srd5a1*: 1.6‐fold; *AKR1C3*: 2.1‐fold; and *HSD17β3*: two‐fold); insulin‐like growth factor (IGF)‐1 signalling [*IGF‐1Ea* (3.5‐fold) and *IGF‐1Ec* (three‐fold)] and myogenic regulatory factors; and the activity of anabolic signalling (e.g. mTOR, AKT, and RPS6; *P* < 0.05) were all up‐regulated with T therapy. Only T up‐regulated mitochondrial citrate synthase activity (*P=*0.03) and transcription factor A (1.41 ± 0.2‐fold, *P=*0.0002), in addition to peroxisome proliferator‐activated receptor‐γ co‐activator 1‐α mRNA (1.19 ± 0.21‐fold, *P=*0.037).

**Conclusions:**

Administration of T adjuvant to RET enhanced skeletal muscle mass and performance, while up‐regulating myogenic gene programming, myocellular translational efficiency and capacity, collectively resulting in higher protein turnover, and net protein accretion. T coupled with RET is an effective short‐term intervention to improve muscle mass/function in older non‐hypogonadal men.

## Introduction

Age‐related declines in muscle mass and strength (sarcopenia)^1^ are associated with mitochondrial abnormalities,^2^ fatigue, lack of energy, loss of libido, decreased sexual performance,^3^ and work capacity.^4^ Many of these symptoms are accompanied by declines in testosterone (T) bioavailability—the ‘andropause'.^5^ Moreover, hypogonadal men exhibit a three‐fold increase in mortality and a five‐fold increased risk of cancer‐related deaths.^6^ Thus, as the ageing populace grows, there remains a need for therapies to counteract the andropause and associated muscle wasting and dysfunction.

Clinically, older men with a total T of <231 ng·dL^−1^ may be offered T therapy^6^ due to established positive effects on fat‐free mass (FFM),^7^ fat mass,^8^ muscle function,^5^ and mitochondrial biogenesis.^9^ Intramuscular T injections offer a safer, more efficacious treatment than oral or transdermal T.^6^ Indeed, dose‐dependent increases in type I and type II fibre areas were found in relation to T injection titrations (25–600 mg·week^−1^) in men >65 years.^10^ In addition, 6 month T injection (~210 mg biweekly) enhanced net protein balance,^5^ myofibrillar protein synthesis (MPS),^11^ FFM,^5^, ^11^ and muscular performance^5^ (albeit through ill‐defined mechanisms). However, the therapeutic efficacy of T injection in older men remains controversial and, likely, non‐beneficial with short‐term physiological dosing (at least in the absence of co‐existing countermeasures). For example, ≤6 week T therapy did not improve MPS, RNA content,^12^ FFM (transdermal 50 mg·day^−1^),^13^ or strength (200 mg biweekly for 12 weeks).^14^, ^15^ Besides administering longer‐term pharmacological doses of exogenous T, interventions that boost endogenous sex hormones, that is, exercise, might be of greater benefit.^16^

In relation to strategies to overcome age‐related muscle dysfunction, the effects of resistance exercise training (RET) on skeletal muscle are well defined. RET promotes muscle anabolism (even in old age^17^, ^18^) and stimulates mitochondrial adaptations^2^; these effects are also associated with enhanced endogenous T in response to acute bouts of RE.^19^ Nonetheless, hypertrophic responses to RET are blunted in older age (vs. younger cohorts), so‐called anabolic resistance, suggesting the need for strategies beyond, or adjuvant to RET,^19^ in order to maximize efficacy. We previously showed that acute RET‐induced systemic induction of endogenous T was related to ensuing muscle hypertrophy in younger men,^20^ suggesting a link between T and muscle mass gains with RET. Moreover, in keeping with a role for T as an adjuvant therapy to RET in older age, 50–100 mg·day^−1^ transdermal T therapy enhanced RET‐induced muscle mass gains over 12 weeks,^21^ while supra‐physiological doses (600 mg·week^−1^) of T adjuvant to RET for 10 weeks increased FFM and strength (in younger men (19–40 years).^22^ Such ‘medium‐term' use of T therapy is indicated for minimizing side effects^23^ of longer‐term T therapy, for example, ≥24 weeks,^24^ and may be useful for pre‐habilitation/rehabilitation (e.g. from bed‐rest). That said, RET interventions of ~12 weeks still engender a major time burden from the perspective of physiotherapy and engender minimal anthropometrical benefits in the absence of adjuvant therapy, even after 24 weeks in older men.^21^

We have previously shown that the majority of muscle mass gains with RET occur early into RET,^20^, ^25^, ^26^ albeit that this is blunted in older individuals. Therefore, in an effort to identify a more time‐efficient and clinically efficient strategy of T therapy and to determine the mechanisms of T therapy adjuvant to RET in relation to anabolic resistance, we investigated the efficacy and cellular mechanisms of short‐term RET, coupled to adjuvant T therapy in older men. In a double‐blind investigation into the effects of 6 weeks of RET with or without T therapy, we determined end‐points relating to muscle mass, function, myogenic regulation, anabolic signalling, and protein turnover.

## Methods

### Study ethics and participants

This study was approved by the University of Nottingham, Faculty of Medicine and Health Sciences Research Ethics Committee (G11082015 SoMS MSGEM), was conducted according to the Declaration of Helsinki, and was pre‐registered at http://clinicaltrials.gov (NCT02152839). Before entry into the study, participants provided written informed consent to participate after all procedures and risks were explained to them. All participants performed activities of daily living and were recreationally active but had not partaken in RET within the previous 12 months. Participants were screened by medical questionnaire, physical examination, routine blood chemistry, and a resting electrocardiogram. Participants who presented with metabolic, respiratory, or cardiovascular disorders or who were prescribed medication (e.g. beta‐adrenergic blocking agents, statins, and anti‐inflammatory drugs) and any other medications that could influence T metabolism were excluded. Of the screened participants, 18 non‐hypogonadal, healthy, normotensive (<140/90) older men with morning serum T concentrations of >230 ng·dL^−1^ (suggested threshold of the lower range of normal T to diagnose hypogonadism^27^) were assigned in a random double‐blinded fashion to receive biweekly injections of either placebo (P, saline *n* = 9, serum T level) or T (Sustanon 250 mg, *n* = 9) (gold standard dosing^28^) over ~6 weeks of whole‐body fully supervised RET. All participants involved in the study were monitored throughout the study for any negative side effects of T injections. No adverse events were reported during or after completion of the study.

### Study conduct

**Figure 1:**
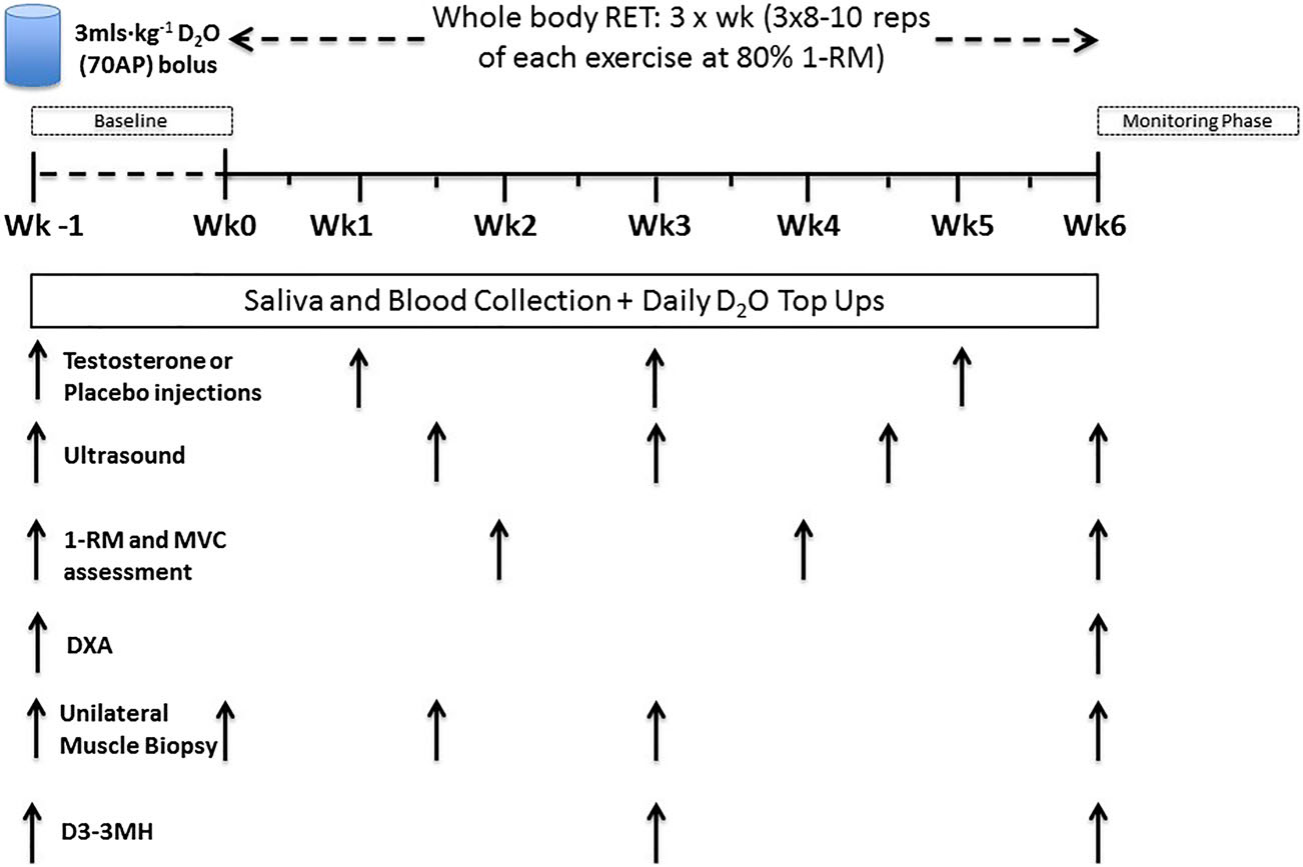
Schematic diagram of the study protocol. 1‐RM, one‐repetition maximum; 3MH, 3‐methylhistidine; DXA, dual‐energy X‐ray absorptiometry; RET, resistance exercise training.

Following baseline measurements of maximal voluntary contraction (MVC) and one‐repetition maximum (1‐RM; on separate days), regardless of group assignment, all participants were further characterized at baseline. This involved collection of fasting blood sampling, muscle ultrasound (Mylab 70; Esaote Biomedica, Italy) of the *m. vastus lateralis* muscle (VL), and a dual‐energy X‐ray absorptiometry (DXA; Lunar Prodigy II, GE Medical Systems, Little Chalfont, UK) scan. Finally, a unilateral muscle biopsy was taken under rested conditions from the VL (*Figure* 1, baseline i.e. Wk ‐1). Given the moderately short half‐life of T (i.e. 4–5 days^29^), it was administered every two‐weeks, and subsequent biopsies were taken at week 0, 1.5, 3, and 6 which were 7, 2, 14, and 7 days after corresponding biweekly T injections, respectively. In order to assess rates of MPS, a basal saliva sample was collected before the muscle biopsy, and the first dose of D_2_O as a bolus of 3 mL·kg^−1^ body weight was consumed by participants after the biopsy. The initial priming dose of D_2_O was followed by daily small‐volume ‘top‐ups' of ~20 mL (calculated from measures of each individual's body water pool turnover). Finally, an injection of T or P was administered by an unblinded clinical research technician. The fully supervised RET protocol then commenced and continued for the next 6 weeks. Additional VL biopsies (60 min after bouts of RE to obtain temporal acute effects of RE across training) and other tests/samples took place intermittently during these 6 weeks (*Figure* 1). All muscle samples were collected under sterile conditions, using the conchotome biopsy technique^30^ with 1% w/v lidocaine as local anaesthetic. Any fat tissue and connective tissue was rapidly dissected out, and muscle was washed in ice‐cold phosphate‐buffered saline and frozen in liquid nitrogen or liquid nitrogen‐cooled isopentane, before storage at −80 °C. Participants were provided 10 mg (D_3_‐methyl)–3‐methylhistidine (3MH) in 100 mL water on three occasions during the study and returned to the research unit (fasted) after 21 h for blood sampling every 2 h (i.e. at 21, 23, and 25 h after 3MH ingestion). A detailed schematic of the study protocol is depicted in *Figure* 1.

### Resistance training procedures and strength assessments

Participants in both groups performed the same whole‐body RET including leg extension, leg press, leg curl, lat pull‐down, shoulder‐press, and bench press (all three sets of 8–10 repetitions at 80% 1‐RM)^31^ three times per week non‐consecutively for 6 weeks. Individuals' 1‐RM^32^ was re‐assessed every 2 weeks before the corresponding training session to maintain intensity with progression. An isokinetic dynamometer (Isocom; Isokinetic Technologies, Eurokinetics, UK) was used to assess isometric knee extensor torque during MVC using two knee joint angles (70 and 80°), with full extension corresponding to 0°. Each individual muscle contraction lasted 4 s, with 30 s rest between contractions and 90 s between knee joint angle assessments. In addition, specific strength (MVC 70°·per LM) was calculated as MVC at 70° divided by leg FFM (LM).

### Muscle architecture by ultrasound and dual‐energy X‐ray absorptiometry‐derived muscle mass

Every 10 days and immediately before corresponding training session, B‐mode ultrasonography (Mylab 70, Esaote Biomedica) with a 100 mm, 10–15 MHz, linear array probe was used for quantification of myo‐architecture. Images were obtained at 50% of the VL length and the mid‐sagittal line while the participant was lying supine on a couch.^33^ To assess fascicle length (Lf), the transducer was aligned with the fascicles to facilitate optimal image capture of the fascicles. The intersection between fascicles and deep tendon aponeurosis and the perpendicular distance between the superficial and deep tendon aponeurosis were used to assess pennation angle (PA) and muscle thickness (MT).^34^ Finally, extended field‐of‐view ultrasonography was used to quantify the cross‐sectional area (CSA) of the quadriceps.^35^ ImageJ software (ImageJ 1.51h) was used to analyse the images.

Before and after the study, DXA (64752, GE Medical Systems‐Lunar Prodigy, USA) was used to determine total FFM, LM, total fat percentage, total fat mass, FFM index (FFM divided by height squared (FFM·height^−2^)), and appendicular FFM (FFM of arms and legs in kilograms divided by square of height in metres). Participants were asked to attend overnight fasting having not performed any heavy physical activity 24 h prior to scanning. For the DXA scan, participants wore loose comfortable clothing with no metal or plastic zippers, buttons, or snaps. Prior to use on participants, a QA block phantom was used to calibrate the system, to ensure optimal measurement. In addition, spine phantoms were run bimonthly to assess the reproducibility and accuracy of the system over time.

### Testosterone enzyme‐linked immunosorbent assay

Venous blood samples were collected into EDTA‐coated tubes intermittently during the study, that is, before injections and prior to individual resistance exercise sessions in the mornings to measure fluctuations in total T concentrations. Blood samples were immediately cold centrifuged at 1750 *g*, with resulting plasma fractions aliquoted and frozen at –80 °C until further analysis. An enzyme‐linked immunosorbent assay (ab108666, Abcam, UK) competitive technique was used to assess the abundance of total T in the plasma of all participants The intra‐assay coefficient of variation was <5%, and the assay sensitivity was 70 ng·dL^−1^; the detection range was 20–1600 ng·dL^−1^.

### Muscle immunohistochemistry

Serial 5‐μm‐thick VL muscle cross‐sections were cut at −20 °C using a cryostat (Leica, CM 1850, Germany), mounted on glass slides, and air dried at room temperature. Determination of fibre‐type‐specific cross‐sectional area was performed using monoclonal antibodies against slow (BA‐F8) and fast myosin (SC‐71) and laminin (D18) (Developmental Studies Hybridoma Bank). Visualization of the primary antibodies was achieved by incubation with Alexa Fluor 488 and 568 goat anti‐mouse secondary antibodies (Invitrogen A/S) *in situ*. Muscle sections were then mounted with Molecular Probes Prolong Gold antifade reagent (Invitrogen A/S). Three major fibre types (I, IIA, and IIX) were determined as previously described.^36^ Fibre area was measured using Sigma Scan Pro 5 software.

### Body water and protein‐bound alanine muscle protein synthesis

To determine the exact volume of D_2_O to be consumed for daily ‘top‐ups', each participant provided saliva for the first 3 days after initial D_2_O consumption. These were processed to determine each participant's body water decay rate, and from this, the amount of D_2_O needed to maintain a steady state over the study period could be calculated. Individuals were then provided with stocks of daily D_2_O ‘top‐ups' (~10% initial bolus dose) with thrice weekly saliva collection for rest of the study period. Wilkinson *et al*.^37^ previously described how body water and muscle protein enrichment were analysed. Briefly, 80–90 μL of saliva was heated in inverted 2 mL autosampler vials for 4 h at 90–100 °C to purify fractions of the body water. The vials were then cooled on ice and before the condensed body water was transferred to a clean autosampler vial for injection. A high‐temperature conversion elemental analyser (Thermo Finnigan, Thermo Scientific, Hemel Hempstead, UK) connected to an isotope ratio mass spectrometer (Delta V advantage, Thermo Scientific) was employed to measure deuterium labelling in saliva (0.1 μL). To assess protein‐bound alanine muscle fraction enrichment, ~40 mg of muscle was homogenized in ice‐cold homogenization buffer to isolate myofibrillar proteins.^20^ Briefly, 10 min rotary mixing was followed by centrifugation at 11 000 *g* for 15 min at 4 °C, the supernatant (sarcoplasmic fraction) was then collected for immunoblotting, and the pellet was resuspended in 500 μL mitochondrial extraction buffer (MEB) and then homogenized by Dounce and centrifuged at 1000 *g* for 5 min at 4 °C. Insoluble collagen was separated following centrifugation from myofibrillar proteins that were solubilized in 750 μL NaOH and subsequently precipitated using 1 M perchloric acid (PCA) then pelleted by centrifugation. Following overnight hydrolysis at 110 °C in a 0.1 M HCl and Dowex H^+^ resin slurry, the amino acids were eluted with 2 M NH_4_OH and dried down. Dried samples were suspended in 60 μL distilled water, 32 μL methanol, and 10 μL pyridine and 8 μL methyl chloroformate with intermittent vortex. The *n*‐methoxycarbonyl methyl esters of the amino acids were then extracted after adding 100 μL chloroform. A molecular sieve was added to remove water for ~20 s before being transferred to vials; incorporation of deuterium into the protein‐bound alanine was determined by gas chromatography–pyrolysis–isotope ratio mass spectrometry (Delta V Advantage, Thermo, Hemel Hempstead, UK).^20^

### Calculation of synthetic fractional rate

MPS was calculated from the deuterium enrichment (APE) in alanine in myofibrillar proteins, using the body water enrichment (APE, corrected for the mean number of deuterium moieties incorporated per alanine, 3.7, and the dilution from the total number of hydrogens in the derivative, i.e. 11) as the precursor labelling between subsequent biopsies. The fractional synthetic rate (FSR) was calculated as follows:

$$\mathrm{FSR}\left(\%\cdot {\mathrm{day}}^{-1}\right)=-\text{In}\left[\frac{1-\left[\frac{({\mathrm{APE}}_{\mathrm{Ala}}}{{\mathrm{APE}}_{P}}\right]}{t}\right],$$

where APE_Ala_ is deuterium enrichment of protein‐bound alanine, APE_P_ is mean precursor enrichment of the body water over the period, and *t* is the time between biopsies. Absolute synthetic rate (ASR) was estimated as follows:

$$\mathrm{ASR}\left(g\cdot {\mathrm{day}}^{-1}\right)=\frac{\mathrm{FSR}}{100}\times \text{Total}\cdot FFM\times \frac{12.4}{100},$$

where alkali‐soluble protein of 12.4% total FFM was assumed.^38^ Absolute protein breakdown rate (ABR) was estimated as follows:

$$\mathrm{ABR}\left(g\cdot {\mathrm{day}}^{-1}\right)=\frac{\mathrm{FBR}}{100}\times \text{Total}\;\mathrm{FFM}\times \frac{12.4}{100},$$

where fractional breakdown rate (FBR) is calculated as FBR = FSR − FGR, with the fractional growth rate (FGR) assumed to be % FFM gain per day over 7 weeks derived by DXA. In addition, the net protein turnover was calculated as follows: ASR − ABR.

### Muscle protein breakdown measures using D_3_–3‐methylhistidine

Precisely, 100 μL of plasma was aliquoted and deproteinized in 1 mL of ice‐cold acetonitrile:methanol (1:1). Following ~1 h of cooling at −20 °C, samples were centrifuged at 17 000 *g* for 20 min at 4 °C. The resulting supernatant was dried and resuspended in 100 μL acetonitrile: ddH_2_O (1:1). Following centrifugation at 17 000 *g*, samples were transferred to autosampler vials for the determination of D_3_–3MH enrichment by liquid chromatography–mass spectrometry (on a Q Exactive Orbitrap, Thermo Hemel Hempstead, UK). The enrichment decay curves were log transformed to determine the decay constant (*k*), which represents the fractional rate of muscle protein breakdown (MPB).^39^

### Muscle RNA, DNA, and protein content

Approximately 15 mg wet weight muscle was used to determine alkaline‐soluble protein (ASP), RNA, and DNA content. Initially, 0.2 M PCA was used to homogenize tissue, followed by centrifugation at 11 680 *g*. Pellets were re‐solubilized in 0.3 M NaOH, and protein contents were quantified by spectrophotometry (NanoDrop Lite, Thermo Scientific). Thereafter, the resulting supernatant was used for RNA quantification at 260 nm by spectrophotometry; the pellet was then heated at 70 °C for 1 h in 2 M PCA to extract the DNA and centrifuged, and DNA was quantified at 268 nm by spectrophotometry.^20^

### Immunoblotting for anabolic/catabolic signalling

Spectrophotometry was used to determine protein concentrations of sarcoplasmic fractions, and samples were diluted with 3× Laemmli loading buffer to 1 mg·mL^−1^, followed by heating at 95 °C for 5 min. Precisely, 10 μg of sample was loaded onto Criterion XT Bis–Tris–12% SDS‐PAGE gels (Bio‐Rad) for electrophoresis at 185 V for 45 min. After electrophoresis, as previously described,^40^ samples were transferred onto polyvinylidene difluoride membranes for 45 min at 100 V. Subsequently, 2.5% low‐fat milk, which was diluted in Tris‐buffered saline Tween 20 (TBST), was used to soak and block polyvinylidene difluoride membranes for 1 h at ambient temperature and then incubated in the following primary antibodies overnight at 4 °C (1:2000 dilution in 2.5% bovine serum albumin in TBST): rabbit phospho‐protein kinase B (Akt)^Ser473^ (#9271), phospho‐mechanistic target of rapamycin (mTOR)^Ser2448^ (#2971), phospho‐mitogen‐activated protein kinase (MEK1/2)^Ser217/221^ (#9121), phospho‐MAP kinase‐activated protein kinase 2 (MAPKAPK‐2)^Thr334^ (#3007), phospho‐ribosomal protein S6 (RPS6)^ser235/236^ (#2211), phospho‐AMP‐activated protein kinase (AMPKα)^Thr172^ (#2531), phospho‐regulatory‐associated protein of mTOR (Raptor)^Ser792^ (#2083), phospho‐forkhead box O3 (FOXO3a)^Ser253^ (#13129) (all from Cell Signaling Technology, Leiden, The Netherlands), muscle‐specific F‐box protein (MAFbx) (#AP2041), muscle RING‐finger protein‐1 (MURF‐1) (#101AP) (both from ECM Biosciences, Versailles, KY, USA), and mouse oxidative phosphorylation (OxPhos) (Abcam, Cambridge, MA, USA). After overnight incubation, membranes were washed 3 × 5 min in TBST and soaked in horseradish peroxidase (HRP)‐conjugated secondary antibody (New England Biolabs; 1:2000 in 2.5% bovine serum albumin in TBST) for 1 h, before 3 × 5 min washes in TBST. In order to quantify band intensity (Chemidoc MP, Bio‐Rad, Hemel Hempstead, UK), membranes were exposed to Chemiluminescent HRP substrate (Millipore Corp., Billerica, MA, USA) for 5 min. Relative arbitrary units were normalized to Coomassie‐stained membranes and to cross gel loading control.

### Gene expression analysis of myogenesis, insulin‐like growth factor‐1 related, and testosterone processing

Approximately 10 mg of muscle was homogenized, with one stainless steel bead (Tissue Lyser II, Qiagen, UK), for 2 min at frequency of 30 s^−1^ in 500 μL TRIzol (Life Technologies/Thermo Fisher Scientific) to isolate total RNA according to the manufacturer's instructions. A high‐capacity cDNA reverse transcription kit (Life Technologies) was used to reverse transcribe 500 ng of total RNA for quantitative reverse transcription PCR. Precisely, 1 μL of 1:10 diluted cDNA was added in each well of 384 optical well plates (Life Technologies). Exon–exon boundary specific primers were mixed with SYBR Select Master Mix (Life Technologies), and RNase‐free water and 6 μL of the mixed solution, as well as 1 μL of each cDNA, were added to each well, with samples run in triplicate. The ViiATM 7 Real‐Time PCR System (Life Technologies) was used according to the following thermal cycling conditions: 2 min at 50 °C, 2 min at 95 °C, and 40 cycles of 15 s at 95 °C and 60 s at 60 °C. The ΔΔCt method was used to quantify target mRNA expression with peptidylprolylisomerase A levels measured to correct for variations in RNA input/cDNA synthesis.^41^ Primer sequences for each of the probed genes are listed in *Table* 1.

**Table 1:** Primer sequences used in PCR

	Forward	Reverse
*RPL13A*	5′‐TAAACAGGTACTGCTGGGCCG‐3′	5′‐CTCGGGAAGGGTTGGTGTTC‐3′
*AR*	5′‐GGTGAGCAGAGTGCCCTATC‐3′	5′‐GCAGTCTCCAAACGCATGTC‐3′
*SRD5A1*	5′‐TACGGGCATCGGTGCTTAAT‐3′	5′‐AATCGCCATTGTACACGCCA‐3′
*AKR1C3*	5′‐GGAGAAGCAGCAGCAAACATT‐3′	5′‐CTTTACTTCTCGGAACCTCTGGA‐3′
*HSD17B3*	5′‐TGTACTCAGCTTCCAAGGCG‐3′	5′‐TATGGGGTCAGCACCTGGAT‐3′
*IGF‐1Ea*	5′‐TCAAATGTACTTCCTTCTGGGTC‐3′	5′‐TAAGGAGGCTGGAGATGTATTGC ‐3′
*IGF‐1Ec*	5′‐AAATCAGCAGTCTTCCAACCC‐3′	5′‐GTGTGCATCTTCACCTTCAAGAAA‐3′
*MHC1*	5′‐ATCTCTACGCCAGGGTCCTTA‐3′	5′‐TTTCGGAGGAAAGGAGCAGC‐3′
*MHCIIa*	5′‐GCCCTTGGAATGAGGCTGAC‐3′	5′‐TGCTGAACTCAGAGGTCCTTGTT‐3′
*Myogenin*	5′‐CCAGGGGATCATCTGCTCACG‐3′	5′‐GGTTTCATCTGGGAAGGCCA‐3′
*Myf‐6*	5′‐CAAGAAAATCTTGAGGGTGCGG‐3′	5′‐TTAGCCGTTATCACGAGCCC‐3′
*C‐Myc*	5′‐AACCACCACCATCCCTGTTTG‐3′	5′‐AAGGCCCCCAGACCCATTTC‐3′
*MEOX‐2*	5′‐TGAAAGACAGGTGAAAGTCTGG‐3′	5′‐ACCAGTTCCTTTTCCCGAGC‐3′
*C‐met*	5′‐ACAGCTGACTTGCTGAGAGG‐3′	5′‐AGGTTTATCTTTCGGTGCCCA‐3′
*PAX7*	5′‐CGGCCAGACTGCTGTTGATTAT‐3′	5′‐GAGTCCCAGCACAGCAGAGT‐3′
*PGC1‐α*	5′‐GAGTCATACTTGCTCTTGGTG‐3′	5′‐GATGATGGAGACAGCTATGGT‐3′
*Tfam*	5′‐TTCGTCCTCTTTAGCATGCTGA‐3′	5′‐CACCGCAGGAAAAGCTGAAG‐3′

*AKR1C3*, aldo‐keto reductase family 1 member C3; *AR*, androgen receptor; *C‐met*, met proto‐oncogene, receptor tyrosine kinase; *HSD17B3*, hydroxysteroid 17‐β dehydrogenase 3; *IGF‐1*, insulin‐like growth factor‐1; *MEOX‐2*, mesenchyme homeobox 2; *MHC*, myosin heavy chain; *Myf‐6*, myogenic factor 6; *C‐Myc*, MYC proto‐oncogene, BHLH transcription factor; *PAX7*, paired box 7; *PGC1‐α*, peroxisome proliferator‐activated receptor‐γ co‐activator 1‐α; *RPL13A*, ribosomal protein L13A; *SRD5A1*, steroid 5 α‐reductase 1; *Tfam*, mitochondrial transcription factor A.

### Mitochondrial citrate synthase activity and DNA copy number

Citrate synthase (CS) activity was measured as described.^42^ Briefly, after homogenization of 3–5 mg muscle in 1% Triton X‐100 buffer, samples were centrifuged at 22 000 *g* for 3 min, and the supernatant was used for further analysis. Thereafter, 300 μL Master Mix containing 28% 0.05 M Tris buffer (pH 7.6), 1.3% 1 mM 5,5′‐dithiobis‐2‐nitrobenzoic acid, 7% acetyl‐coenzyme A (1.36 mg·mL^−1^), 0.8% oxaloacetate (9.88 mg·mL^−1^), and 63% ddH_2_O was measured at 412 nm as the blank. Finally, 20 μL of separated supernatant was used to measure the maximum rate of reaction (*V*_max_), compared with whole protein content. To quantify relative mitochondrial DNA (mtDNA) copy number, the extraction of genomic and mtDNA from muscle was performed using a Qiagen QIAamp® DNA Mini Kit, according to the manufacturer's recommendations. Briefly, the procedure involved tissue lysis in a proteinase K buffer, incubation for 3 h at 56 °C to digest myofibrils followed by centrifugation of the lysates through silica membrane‐based nucleic acid purification columns and subsequent elution of the mtDNA and gDNA. The quality and quantity of DNA was assessed by measurement at 260, 280, and 230 nm. The expression level of markers of gDNA and mtDNA used to evaluate their abundance was accomplished by using TaqMan probe real‐time PCR. The TaqMan probe design for the detection of gDNA levels was based on interrogation of the intron sequence spanning between exons 3 and 4 of the genomic hydroxymethylbilane synthase (HMBS) gene to avoid mRNA amplification. The probe design for detection of mtDNA levels was based on interrogation of a stable fragment of the mtDNA loop, namely, the mitochondrially encoded NADH:ubiquinone oxidoreductase core subunit 1 (ND1). The 2^−ΔCt^ formula, where Δ = Ct_ND1_ − Ct_HMBS_, was used to express relative mtDNA copy number.

### Statistical analyses

Data are expressed as mean ± standard error of the mean, while normality of distribution was examined using the Kolmogorov–Smirnov test. In addition, analysis of covariance using baseline values for each outcome as a covariate and repeated measures analysis of variance (time) with one between‐subject factor (group) were used to compare the changes during the RET programme both within and between the two (P vs. T) groups. In addition, independent *t*‐tests were used for comparing fold change between the two groups. Cohen's effect sizes (ES) were also calculated for significant data. ES of 0 to <0.20 were considered ‘trivial', 0.20 to <0.50 were considered ‘small' in magnitude, 0.50 to <0.80 were considered ‘medium', and ≥0.80 were considered ‘large'.^43^ Where significant differences were found using repeated measures analysis of variance, a Bonferroni *post hoc* test was applied for multiple comparisons. The correlation was assessed using Pearson's product moment correlation coefficient and intraclass correlation coefficient (ICC) was used to test reliability of DXA and ultrasound‐related outputs. The significance level was defined as *P* ≤ 0.05, and all of the statistical analyses were performed using GraphPad Prism 7.01 (La Jolla, CA, USA).

## Results

**Figure 2:**
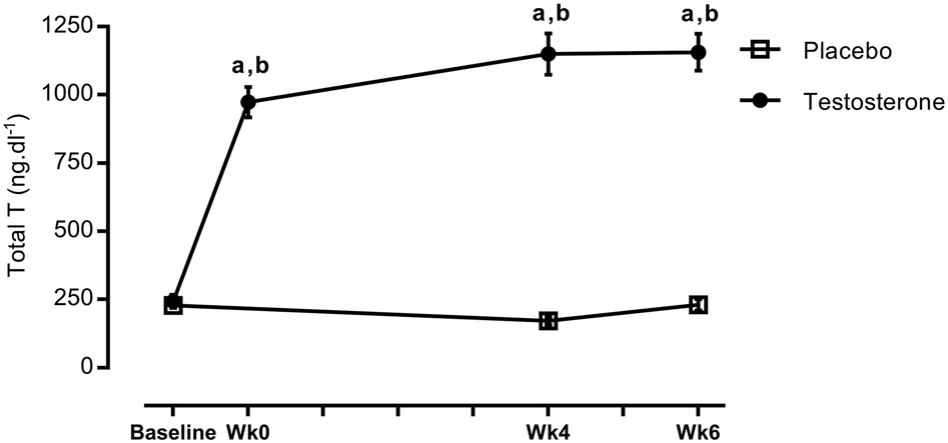
Time course of changes in total testosterone. ^a^Significantly different from baseline, *P* < 0.05; ^b^significantly different between two groups, *P* < 0.05.

Physiological characteristics of participants are shown in *Table* 2. In accordance, only the T study group significantly increased weight (*P=*0.006, 95% confidence interval (CI) = 81–83, ES = 0.19) and body mass index (*P=*0.006, 95% CI = 26–26.6) after 6 weeks of RET, primarily through significant gains in FFM in the T group vs. baseline (see the Body composition section). Further, total plasma T concentrations in the T therapy group were significantly higher than in the P group at all time‐points after baseline (*P* < 0.05), that is, 234.9 ± 7 ng·dL^−1^ at baseline, and achieved 973 ± 54 at 0 week and 1149 ± 75 ng·dL^−1^ after 5 weeks in T group (95% CI = 1044–1232). Plasma T concentrations during the study are shown in *Figure* 2.

**Table 2:** Participant characteristics

	T (*n* = 9)		P (*n* = 9)	
	Baseline	Week 6	Baseline	Week 6
Age (years)	69.7 (0.8)	––	69.5 (1.3)	––
Height (m)	1.75 (0.02)	––	1.76 (0.02)	––
Weight (kg)	78.6 (3.1)	80.4 (3.2)^a^	81.8 (4.3)	82.4 (4.1)
BMI (kg·m^−2^)	25.7 (1)	26.2 (1)^a^	26.5(1.1)	26.7 (1.1)

P, placebo; T, testosterone.

Values are means (standard error of the mean).

a Significantly different from baseline, *P* < 0.05.

### Body composition

**Figure 3:**
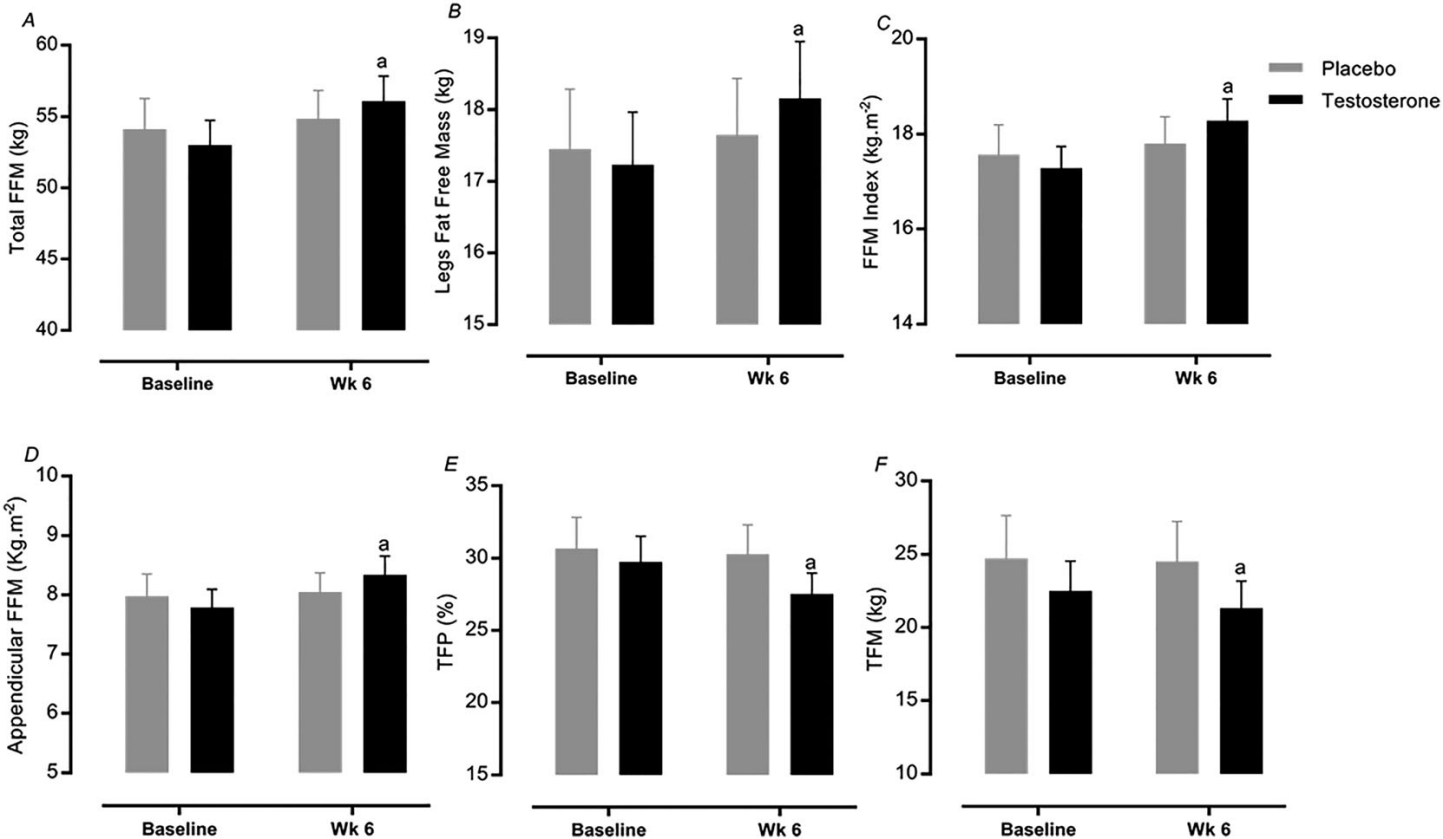
Muscle mass and body composition changes. (A–F) Values are means ± standard error of the mean. ^a^Significantly different from baseline, *P* < 0.05. FFM, fat‐free mass; TFP, total fat percentage; TFM, total fat mass.

Total FFM (ICC = 0.94) increased significantly following 6 week RET in T (53.0 ± 1.7 to 56.0 ± 5.2 kg, *P=*0.007, 95% CI = 55.5–57.6, ES = 0.45, *Figure* 3A) but not in P (54.1 ± 6.3 to 54.8 ± 5.8 kg, 95% CI = 53.2–55.4). Likewise, only T augmented LM (17.2 ± 0.7 to 18.1 ± 0.8 kg, 95% CI = 17.8–18.6 vs. 17.4 ± 0.8 to 17.6 ± 0.7 kg, 95% CI = 17.1–17.9, *P=*0.02, ES = 0.30, ICC = 0.96, *Figure* 3B), appendicular FFM (7.8 ± 0.3 to 8.3 ± 0.3, 95% CI = 8.2–8.5 vs. 7.9 ± 0.3 to 8.0 ± 0.3, 95% CI = 7.7–8.1, *P=*001, ES = 0.54, ICC = 0.94, *Figure* 3D), and FFM index (17.2 ± 0.4 to 18.2 ± 0.4 kg·m^−2^, 95% CI = 18.1–18.7 vs. 17.5 ± 0.6 to 17.8 ± 0.5 kg·m^−2^, 95% CI = 17.3–18.1, *P=*0.005, ES = 0.42, ICC = 0.92, *Figure* 3C) after RET. In addition, both total fat percentage (29.7 ± 1.7 to 27.5 ± 1.4%, 95% CI = 27–28.8 vs. 30.6 to 30.2%, 95% CI = 29–30.7, *P=*0.004, ICC = 0.96, ES = 0.43) and total fat mass (22.5 ± 2.0 to 21.3 ± 1.8 kg, 95% CI = 21.6–23.1 vs. 24.7 ± 2.8 to 24.5 ± 2.7 kg, 95% CI = 22.8–24.2, *P=*0.02, ICC = 0.97, ES = 0.29) decreased significantly in T but not in P (*Figure* 3E and 3F).

### Muscle architecture

**Figure 4:**
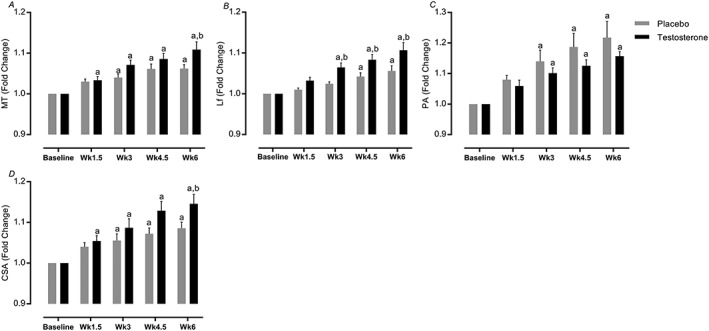
(A–D) Fold change in muscle architecture. Values are means ± standard error of the mean. ^a^Significantly different from baseline, *P* < 0.05; ^b^significantly different between two groups, *P* < 0.05. CSA, cross‐section area; Lf, fascicle length; MT, muscle thickness; PA, pennation angle.

Over the RET period, T and P both exhibited increases in MT (2.3 ± 0.07 to 2.6 ± 0.04 cm, 95% CI = 2.5–2.6, *P* < 0.0001, ES = 0.83 vs. 2.3 ± 0.1 to 2.4 ± 0.09 cm, 95% CI = 2.4–2.5, *P* < 0.0001, ICC = 0.81, ES = 0.47), Lf (7.1 ± 0.2 to 7.9 ± 0.2 cm, 95% CI = 7.9–8.2, *P* < 0.0001, ES = 0.84 vs. 7.7 ± 0.1 to 8.1 ± 0.1 cm, 95% CI = 7.7–8, *P* < 0.0001, ES = 0.73), PA (20.0 ± 0.8 to 23.1 ± 0.7°, 95% CI = 21.2–22.6, *P* < 0.0001, ES = 1.2 vs. 17.0 ± 1.2 to 20.3 ± 0.9°, 95% CI = 20.7–22.1, *P* < 0.0001, ES = 0.88), and CSA of the quadriceps (61.4 ± 3.1 to 69.9 ± 2.6 cm^2^, 95% CI = 66.2–70.9, *P* < 0.0001, ES = 0.93 vs. 58.8 ± 4.1 to 64 ± 4.8 cm^2^, 95% CI = 62.9–67.7, *P* < 0.0001, ES = 0.41, ICC = 0.89). The majority of increases were to a greater extent in the T group and with significant differences between groups in MT (*P=*0.01), Lf (*P=*0.001), and CSA (*P=*0.04) (*Figure* 4A–C).

### Fibre‐type cross‐sectional area

**Figure 5:**
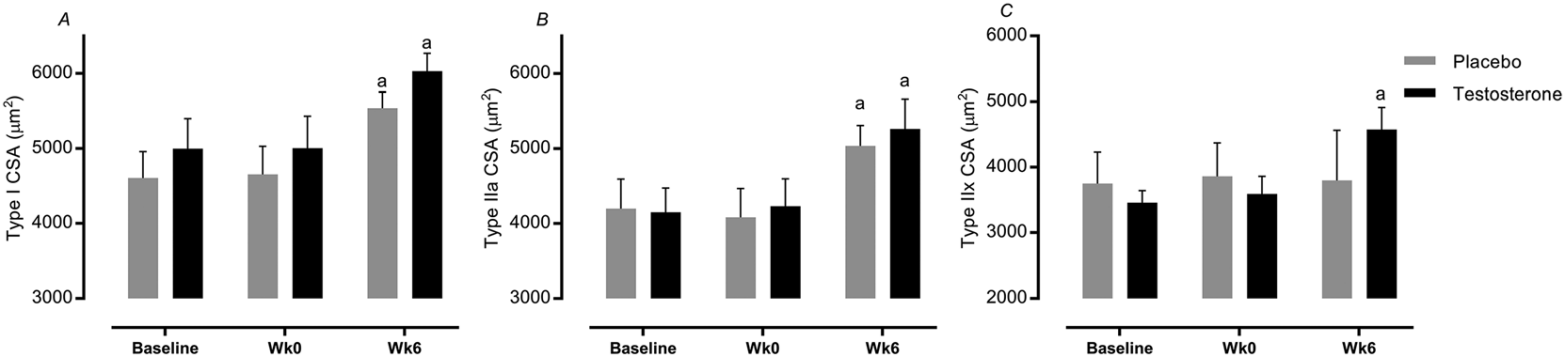
(A–C) Time course of changes in fibre‐type cross‐section area (CSA). Values are means ± standard error of the mean. ^a^Significantly different from baseline, *P* < 0.05; ^b^significantly different between two groups, *P* < 0.05.

Type I and IIa CSA increased in both the T and P groups after RET (*P* < 0.05), but only T augmented type IIx (3455 ± 187 to 4576 ± 338 μm^2^, 95% CI = 3814–5659 vs. 3751 ± 478 to 3797 ± 767 μm^2^, 95% CI = 2560–4630, *P* = 0.02, ES = 0.64, *Figure* 5A–C).

### Muscular performance

**Figure 6:**
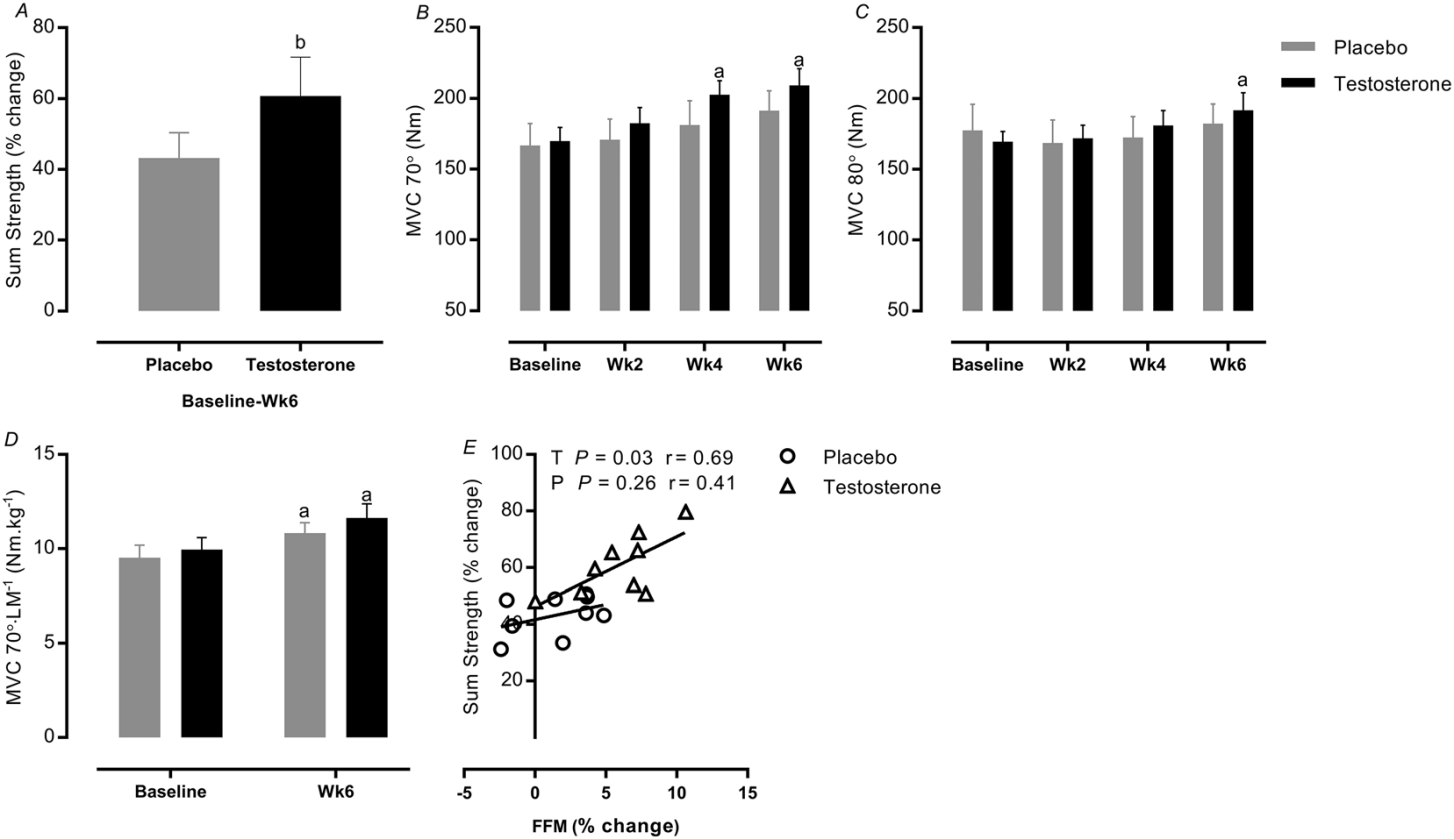
Time course of changes in (A) sum strength and (B–D) muscle strength and (E) correlation between fat‐free mass (FFM) and sum strength. Values are means ± standard error of the mean. ^a^Significantly different from baseline, *P* < 0.05; ^b^significantly different between two groups, *P* < 0.05. LM, legs fat‐free mass; MVC, maximal voluntary contraction.

In line with higher levels of T (*P* < 0.0001, *Figure* 2), only the T group showed a greater increase in static strength after RET, for example, MVC at 70° (169.9 ± 9.6 to 209 ± 11.8 Nm, 95% CI = 11.5–66.6, *P=*0.002, ES = 1.3, *Figure* 6B) and 80° (169.4 ± 7.3 to 191.7 ± 12.5 Nm, 95% CI = 3.8–40.5, *P=*0.01, ES = 1.01, *Figure* 6C) vs. baseline. Furthermore, dynamic strength (sum 1‐RM across all six exercises) was augmented into a greater extent in the T group vs. P (60.8 ± 3.6 vs. 43.25 ± 2.4%, 95% CI = T: 54.2–67.7 vs. P: 36.7–49.7, *P=*0.0009, ES = 0.96, *Figure* 6A). There were similar increases in specific strength (force per unit area) across the groups (95% CI = T: 10.4–12.4; P: 10.1–11.9, ES = T: 0.88; P: 0.65, *P* < 0.05, *Figure* 6D). Finally, FFM gains were correlated with sum strength gains in the T study group but not in the P group (*Figure* 6E).

### Muscle protein synthesis and (extrapolated) breakdown

**Figure 7:**
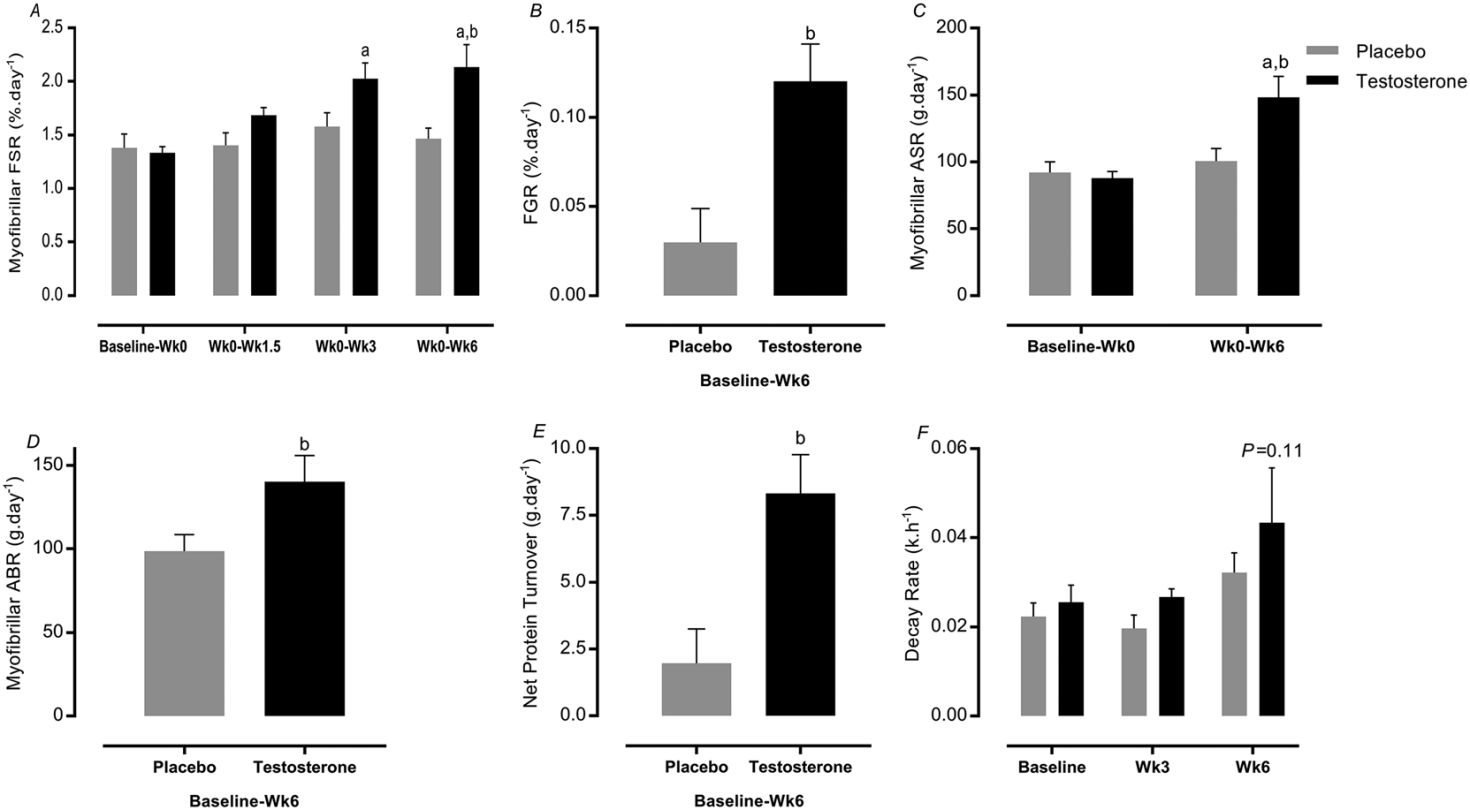
Muscle protein synthesis, fractional growth rate (FGR), absolute synthetic rate (ASR), absolute breakdown rate (ABR), and protein turnover. Values are means ± standard error of the mean. ^a^Significantly different from baseline, *P* < 0.05; ^b^significantly different between two groups, *P* < 0.05; *P* < 0.11 Week 6 vs. baseline in testosterone group. FSR, fractional synthetic rate.

There was a significant difference in cumulative MPS between T and P during RET (2.13 ± 0.21 vs. 1.34 ± 0.13%·day^−1^, 95% CI = 1.7–2.4 vs. 1.1–1.8, *P=*0.0009, ES = 0.75), in line with findings of elevated FGR in T compared with P (0.12 ± 0.02 vs. 0.03 ± 0.01%·day^−1^, 95% CI = 0.07–0.16 vs. −0.12 to 0.07, *P=*0.005, ES = 0.85) (*Figure* 7A and 7B). Furthermore, ASR was significantly increased as a result of RET in T (87.8 ± 5.1 to 148.5 ± 15.3 g·day^−1^, 95% CI = 120–176, *P=*0.02, ES = 0.31) but not in P (84.7 ± 4.7 to 92.2 ± 11.3 g·day^−1^, 95% CI = 72–128, *P* > 0.05). In addition, estimated ABR was significantly higher in T than P during RET (140.2 ± 15.8 vs. 90.2 ± 11.7 g·day^−1^, 95% CI = 112–168 vs. 70–126, *P=*0.02, ES = 0.55). Finally, when comparing ASR and ABR, net protein turnover in the T group was higher than in the P group (8.3 ± 1.4 vs. 1.9 ± 1.2 g·day^−1^, 95% CI = 5.4–11.2 vs. −0.9 to 4.8, *P=*0.004, ES = 0.86, *Figure* 7C–E). Using D_3_–3‐MH, there was no significant difference in MPB between T and P at 6 weeks over the 4 h sampling period (T: 0.043 ± 0.012 vs. P: 0.032 ± 0.004 *k*·h^−1^, *P=*0.5) (*Figure* 7F).

### Muscle alkaline‐soluble protein, DNA, and RNA content

There were no changes in ASP or DNA concentrations per wet weight muscle (μg·mg^−1^) in T or P with RET; nonetheless, total RNA content (95% CI = T: 2.1–2.5; P: 1.6–2.1, *P=*0.002, ES = 0.78) and RNA:DNA ratio, a reflection of ribosomal capacity for protein synthesis, only increased in the T group (95% CI = T: 1.1–1.2; P: 0.7–1, *P=*0.002, ES = 0.83). Similarly, RNA:ASP ratio, primarily a measure of ribosomal capacity,^20^ increased in T (95% CI = 17.8–23.5, *P=*0.002, ES = 0.24) but not in P (*P=*0.6, 95% CI = 15.2–20.9) over the RET phase. Finally, the ratio of ASP:DNA, a measure of cell size, did not change in either group (*Table* 3).

**Table 3:** Muscle protein, RNA, DNA, and related ratios

	Placebo				Testosterone			
	Baseline	Week 0	Week 3	Week 6	Baseline	Week 0	Week 3	Week 6
ASP content (μg·mg^−1^ ww)	110.5 (3.9)	108.5 (2.8)	107.4 (4.9)	106.9 (4.5)	118.2 (3.7)	115.3 (3.4)	118.2 (2.7)	118.5 (4.6)
RNA content (μg·mg^−1^ ww)	1.6 (0.1)	1.8 (0.1)	2.1 (0.1)	1.9 (0.1)	1.7 (0.1)	2.1 (0.1)	1.8 (0.1)	2.3 (0.1)^a^
DNA content (μg·mg^−1^ ww)	2.2 (0.1)	2.3 (0.1)	2.2 (0.1)	2.2 (0.1)	2.2 (0.1)	2.2 (0.1)	2.1 (0.1)	2.1 (0.1)
RNA:DNA (ratio)	0.7 (0.1)	0.7 (0.1)	0.9 (0.1)	0.8 (0.1)	0.7 (0.1)	0.9 (0.1)	0.8 (0.1)	1.1 (0.1)^a^
RNA:ASP (ratio)	15.3 (0.5)	17.1 (0.8)	19.9 (1.7)	18.1 (0.9)	14.6 (0.9)	17.8 (1.5)	15.5 (0.6)	20.7 (1.5)^a^
ASP:DNA (ratio)	50.7 (3.6)	46.1 (1.7)	49.2 (3.8)	47.9 (3.1)	54.2 (2.4)	52.6 (2.4)	56.5 (2.4)	56.6 (5.2)

ww, wet weight.

Values are means (standard error of the mean).

a Significantly different from baseline, *P* < 0.05.

### Intramuscular signalling pathways

**Figure 8:**
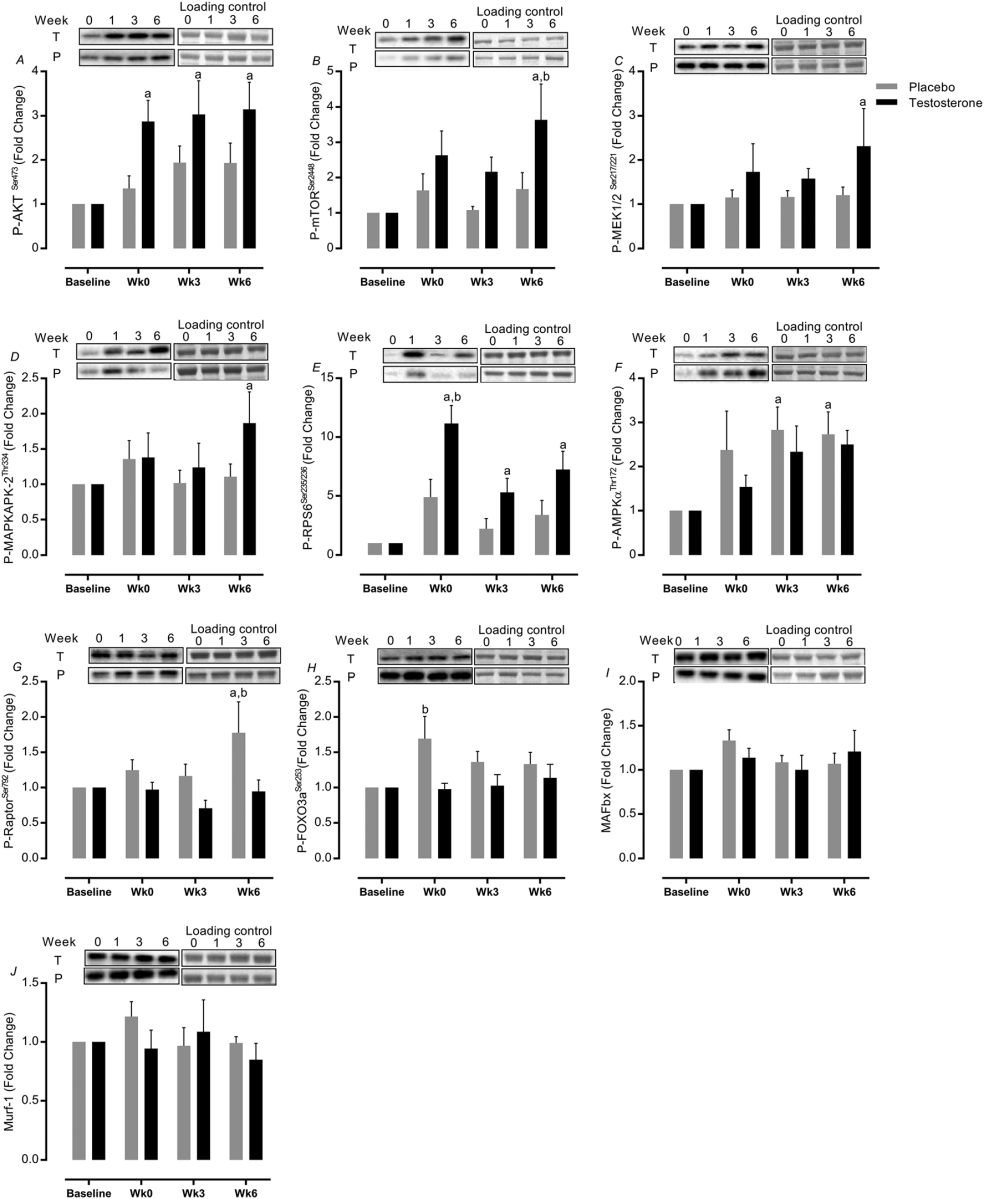
Intramuscular signalling pathways. Values are means ± standard error of the mean. ^a^Significantly different from baseline, *P* < 0.05; ^b^significantly different between two groups, *P* < 0.05.

Acute exercise‐induced anabolic signalling was quantified 60 min after the first (0 week), ninth (3 weeks), and 18th (6 weeks) resistance exercise sessions. We observed significant increases in the acute phosphorylation of AKT^Ser473^ (95% CI = 2–4.2 vs. 0.7–3, *P=*0.0006, ES = 0.66), mTOR^Ser2448^ (95% CI = 1.9–5.3 vs. 0.1–3.3, *P=*0.001, ES = 0.0.64), MEK1/2^Ser217/221^ (95% CI = 1.1–3.6 vs. −0.1 to 2.5, *P=*0.02, ES = 0.43), MAPKAPK‐2^Thr334^ (95% CI = 1.1–2.6 vs. 0.3–1.8, *P=*0.03, ES = 0.32), and RPS6^ser240/244^ (95% CI = 4.2–10.2 vs. 0.3–6.3, *P=*0.001, ES = 0.44) in T (6 weeks vs. baseline) but not in P. In contrast to this, the P group increased activation of AMPKα^Thr172^ (95% CI = 1.5–3.4 vs. 1.8–3.6, *P=*0.02, ES = 0.25) and Raptor^Ser792^ (95% CI = 0.24–1.6 vs. 1.1–2.4, *P=*0.01, ES = 0.84) after 6 weeks RET. Furthermore, FoxO3a^Ser253^ phosphorylation (*P=*0.01) was increased only after the first exercise bout in the P group. The relative abundance of MAFbx and MURF‐1 was unchanged throughout the study (*Figure* 8).

### Muscle gene expression

**Figure 9:**
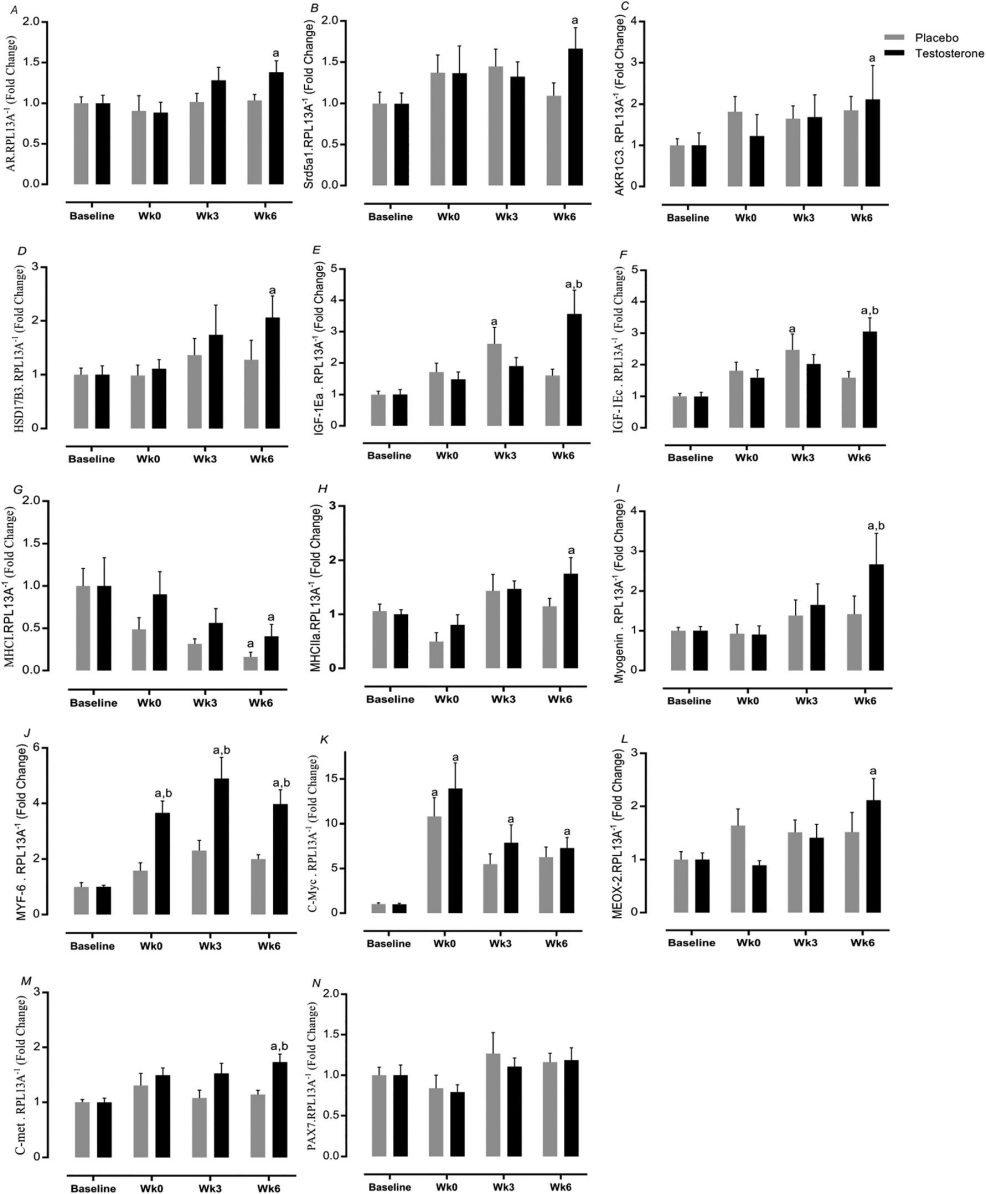
Muscle gene expression. Changes in testosterone and placebo in (A–D) testosterone metabolism gene expression, (E, F) anabolism, (G, H) fibre type, and (I–N) myogenic from baseline to Week 6. Values are means ± standard error of the mean. ^a^Significantly different from baseline, *P* < 0.05; ^b^significantly different between two groups, *P* < 0.05.

The expression of a number of genes involved in T metabolism was augmented in T vs. baseline but not in the P group, that is, androgen receptor (*AR*) (1.4 ± 0.1‐fold change, 95% CI = 1.2–1.5 vs. 0.84–1.2, *P=*0.01, ES = 0.71), *Srd5a1* (1.6 ± 0.2‐fold change, 95% CI = 1.3–1.9 vs. 0.76–1.4, *P=*0.01, ES = 0.7), *HSD17β3* (2 ± 0.4‐fold change, 95% CI = 1.3–2.7 vs. 0.5–1.9, *P=*0.03, ES = 0.33), and *AKRIC3* (2.1 ± 0.8‐fold change, 95% CI = 1.2–3.1 vs. 0.9–2.7, *P=*0.01, ES = 0.14, *Figure* 9A–D) increased after 6 week RET only in T. In addition, *IGF‐1Ea* (3.5 ± 0.07‐fold change, 95% CI = 2.3–4.7 vs. 0.4–2.8, *P* < 0.0001, ES = 0.63) and *IGF‐1Ec* (3 ± 0.4‐fold change, 95% CI = 2.3–3.7 vs. 0.8–2.2, *P* < 0.0001, ES = 0.83) expression increased only in T after 6 week RET (*Figure* 9E and 9F). While mRNA expression of *MHCI* decreased in both groups (*P* < 0.05), the expression of *MHCIIa* was augmented only in T (1.72 ± 0.3‐fold change, 95% CI = 1.2–2.2 vs. 0.6–1.7, *P=*0.02, ES = 0.33) (*Figure* 9G and 9H). T coupled with RET augmented myogenesis‐related gene expression, that is, *Myogenin* (2.6 ± 0.7‐fold change, 95% CI = 1.3–4.1 vs. 0.1–2.7, *P=*0.02, ES = 0.53), *C‐Myc* (7.2 ± 1.1‐fold change, 95% CI = 5.3–9.1 vs. 4.3–8.3, *P=*0.04, ES = 0.28), *Myf‐6* (3.9 ± 0.5‐fold change, 95% CI = 3.1–4.7 vs. 1.1–2.8, *P* < 0.0001, ES = 0.92), *MEOX‐2* (2.1 ± 0.4‐fold change, 95% CI = 1.3–2.8 vs. 0.7–2.2, *P=*0.01, ES = 0.19), *C‐met* (1.7 ± 0.1‐fold change, 95% CI = 1.4–1.9 vs. 0.8–1.4, *P=*0.003, ES = 0.89) but not *PAX‐7* (*P* > 0.05) as a marker of satellite cell proliferation (*Figure* 9I–N).

### Mitochondrial oxidative phosphorylation capacity

**Figure 10:**
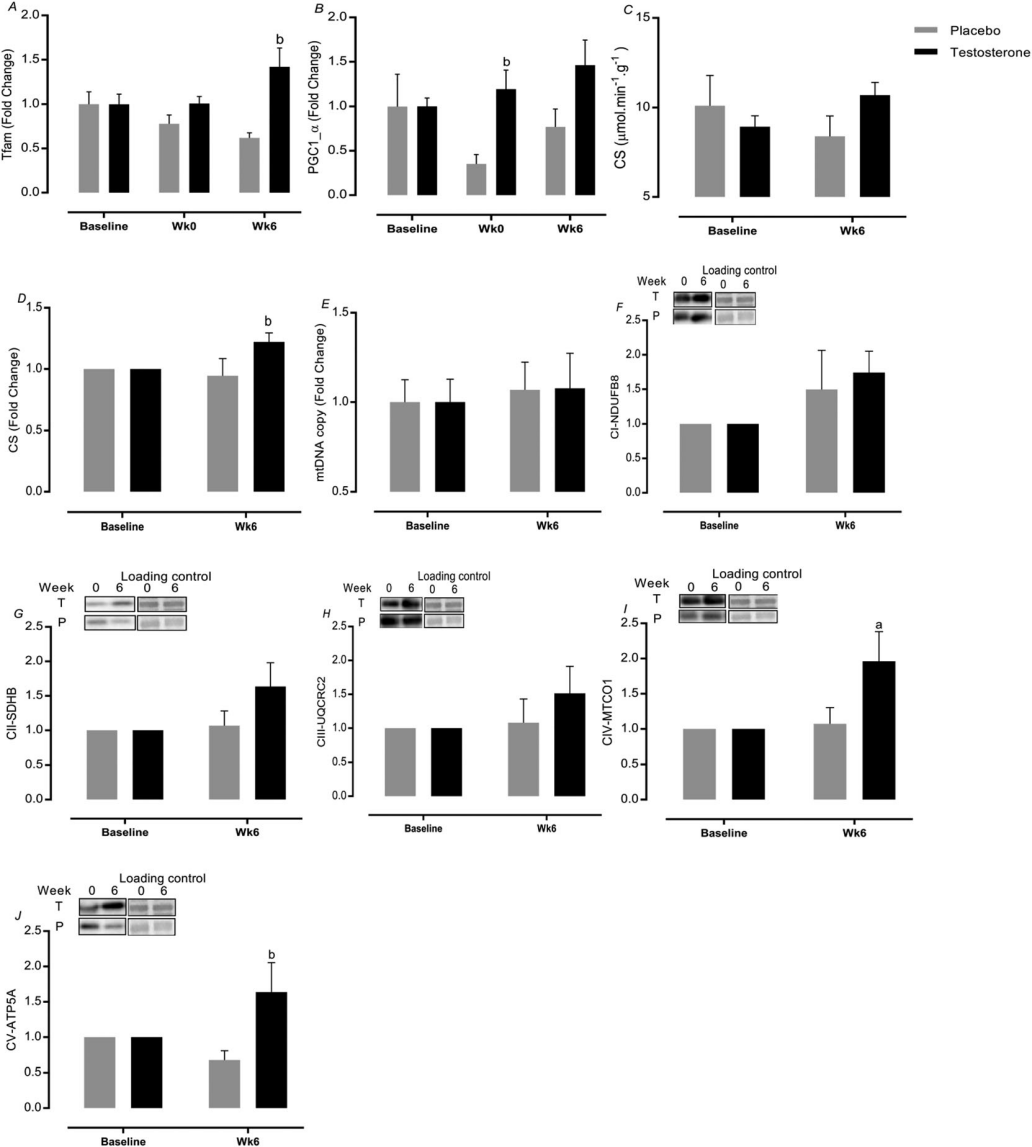
Mitochondrial oxidative phosphorylation capacity markers. Values are means ± standard error of the mean. ^a^Significantly different from baseline, *P* < 0.05; ^b^significantly different between two groups, *P* < 0.05.

Testosterone augmented mitochondrial transcription factor A (*Tfam*) expression at 6 weeks (1.4 ± 0.2‐fold change, 95% CI = 1.1–1.7 vs. 0.2–0.9, *P=*0.0002, ES = 0.91), whereas expression of peroxisome proliferator‐activated receptor‐γ co‐activator 1‐α (PGC1‐α) was increased only after the first bout of exercise (1.2 ± 0.2‐fold change, 95% CI = 1.1–1.8 vs. 0.3–1.1, *P=*0.037, ES = 0.7, *Figure* 10A and 10B). In addition to increased mitochondrial density (assessed by relative change of CS activity) in the T group (1.22 ± 0.07‐fold change, 95% CI = 0.9–1.4 vs. 0.7–1.1, *P=*0.03, ES = 0.29, *Figure* 10D), only the T group showed enhanced protein levels of OxPhos complex (C)‐IV (95% CI = 1.2–2.6 vs. 0.3–1.7, *P=*0.02, ES = 0.41) and V (95% CI = 0.9–2.2 vs. 0.1–1.3, *P=*0.04, ES = 0.23), while there was a trend in C‐II (*P=*0.08) but not in C‐I (*P=*0.24) or C‐III (*P=*0.3) (*Figure* 10F–J). There was no impact of RET or T therapy upon mtDNA copy number (*P* > 0.05, *Figure* 10E).

## Discussion

There is a great deal of interest in identifying interventions that will combat muscle wasting in older age. Herein, we elucidated that just 6 week whole‐body RET coupled with the exogenous provision of T had significant positive effects on FFM, and muscular performance via inducing net protein accretion through anabolic pathways, thus offsetting age‐related deficits in adaptive responses to RET.^20^

The role of T in regulating muscle gains when coupled with RET remains somewhat contentious. For instance, Ahtiainen *et al*.^44^ reported that 12 month RET increased FFM without altering endogenous T levels, concluding that changes in endogenous T levels did not impact RET‐induced muscular adaptations.^44^ However, in another study, suppression of endogenous T using Zoladex blunted muscle adaptations to 8 week RET in younger men^45^, suggesting that endogenous T levels are, at very least, permissive in regulating hypertrophic adaptations to RET. Therefore, given established links between ageing and muscle loss, declines in T responses to acute RE,^20^ and age‐related anabolic resistance to RET,^20^ we postulated that short‐term T adjuvant to RET would be of benefit. Consistent with this thesis, it was previously reported that exogenous T therapy adjuvant to RET for longer periods (10–12 weeks) led to greater muscle mass gains^46^, ^47^ than with RET alone. Our present albeit shorter‐term data also showed that RET adjuvant to T led to significant increases in whole‐body and appendicular FFM. Moreover, RET‐induced gains in MT, Lf, PA, and CSA in VL and quadriceps illustrate that T therapy augmented local thigh muscle remodelling, mainly by increasing Lf (number of sarcomeres in series^48^) rather than increases in PA; remodelling also associated with T‐specific hypertrophy of type IIX fibres. Previous studies in younger adults have shown that the hypertrophic response to T occurs in both slow and fast muscle fibres^49^, ^50^, ^51^ (although these studies were over longer periods). Considering previously described preferential loss of fast fibres during ageing,^52^, ^53^ our data suggest that exogenous provision of T to healthy non‐hypogonadal older men can be considered as a therapy preventing atrophy—and perhaps loss—of these muscle fibres.

Because muscular performance is the culmination of neural and muscle structural elements and is the most important attribute to maintaining mobility, we determined the role of exogenous T in relation to aspects of muscle function. In doing so, we established that the T group developed greater static (MVC) and sum dynamic (1‐RM) strength gains (in line with greater muscle mass gains that strongly correlated with strength improvement). Interestingly, previous work has shown that there may be a neural component to the effects of T, for example, influencing neurotransmitter synthesis,^54^ leading to reduced force fluctuations^55^ and inducing recruitment of larger/faster motor units (fast twitch fibres).^56^ Nonetheless, our MVC per LM ‘specific force' data illustrate that muscle mass gains were associated with strength‐related improvements; given the strong correlation between FFM gains and strength improvement in T group, we can thus conclude that RET‐induced FFM gains were a major component of improved muscle performance in both groups. Physiological endogenous T levels are correlated with RET‐induced gain of FFM and strength,^57^ perhaps explaining blunted adaptation to RET in older vs. younger men.^20^, ^58^ Dose–response studies reported that higher doses (supra‐physiological doses; i.e. 600 mg weekly) of T result in graded increments in FFM and strength than lower doses (physiological doses) in men, indicating that efficiency of the T therapy is associated with the higher T doses.^22^, ^59^, ^60^ Thus, the ‘moderately' supra‐physiological T levels^61^ in our study reveals that short‐term T supplementation was able to ‘overcome' previously observed anabolic resistance to RET^20^ in relation to muscle growth. However, further studies are needed to fully address the physiological vs. supra‐physiological prerequisites to efficacy.

In order to assess the mechanisms underlying the ergogenic effects of T therapy adjuvant to RET in older men, we for the first time quantified cumulative rates of myofibrillar protein synthesis over the 6 weeks of RET. In doing so, we demonstrated that T augmented MPS during RET while concomitantly increasing (estimated) FBR^62^ (which is supported by the 3MH data) and crucially with net balance calculations illustrating MPS > MPB. MPB is an important metabolic component of muscle remodelling and protein turnover increase with RET but to a lesser extent than MPS.^63^ The logical extrapolation of a sustained increase in net muscle protein deposition after RET is an increase in FFM and strength,^5^ entirely in line with our mass and strength gain data. Links between T and MPS have been previously established. For instance, decreased muscle mass with castration‐induced androgen withdrawal suppressed myofibrillar protein synthesis in mice.^64^ In addition, 6 months^11^ and 4 weeks^65^ of T administration increased MPS measured acutely in older men. Further, T administration led to increases in MPS without concomitant increases in inward amino acid transport,^66^ indicating increased efficiency of reutilization of amino acids from MPB with T.^66^ Interestingly, it was suggested that T administration may improve net protein balance via decreasing MPB, rather than an increasing MPS.^67^ However, we show that T coupled to RET increased net protein accretion despite elevation of both MPS and MPB, because increases in MPS > MPB.

In order to explore the mechanisms underpinning increases in MPS, we investigated aspects regulating the translational ‘efficiency' of ribosomes^68^ corresponding to the translational rate per ribosome.^69^ To evaluate this, we determined acute RE‐induced phosphorylation within MAPKAPK‐2 (target of MAPK pathway), MEK1/2 (upstream components in ERK1/2 cascade),^70^ and mTORC1^20^ pathways, across the duration of the 6 week RET. It is noteworthy from past work that activation of many of these pathways is blunted in older age^20^, ^70^, ^71^ and that in the present study, T administration could reverse these impairments. Consistently, there is a general blunted activation of mTORc1,^71^ ERK1/2,^70^ and MAPKs^70^ presumably due to lesser bioavailability of T.^64^, ^72^ In support of this, T therapy augmented IGF‐1/Akt/mTOR signalling activity (which regulates MPS^64^), presumably explaining enhanced mass and functional gains and suggesting that T levels, at least permissively, regulate muscle adaptations. In addition to increases in translational efficiency, another facet that is blunted with age^20^ and potentially impacts ‘capacity' for increasing protein synthesis and net protein balance and is critical for anabolic potential and hypertrophy is ribosomal RNA content.^73^ In the present study, exogenous T therapy coupled with RET increased total RNA content per ‘cellular unit' (RNA:DNA ratio), an indicator of ribosomal abundance,^74^ and total RNA:ASP ratio, as an index of synthetic capacity, thereby offsetting age‐related deficits.^75^ Although we observed only subtle (non‐significant) increases in ASP:DNA ratio, an indicator of myonuclear domain size,^74^ despite there being robust increases in muscle mass (DXA, ultrasound, and histology), this is to be expected because of the maintenance of the myonuclear domain size beyond which additional hypertrophy can only be realized by addition of myonuclei.^68^

No study has previously examined the effect of RET adjuvant to T in relation to muscle steroid‐metabolism handling in older human muscle. In our study, T therapy augmented AR mRNA and steroidogenic enzyme expression perhaps resulting in higher muscle T processing capacity, presumably due to infiltration of T from the circulation^76^ and processing by 5‐α reductase. Furthermore, the AR^77^ not only alters mRNA expression of thousands of target genes 78 but also is associated with fibre‐type CSA increases,^79^ as well as triggering anabolic kinase signalling, that is, ERK, PI3K, and Akt,^72^ suggesting transcriptional links to augmented MPS, FFM, and strength in the T group in the present study and others.^26^, ^79^, ^80^, ^81^ In addition to increases in T metabolism‐related mRNA expression, we demonstrated that myogenic and growth factor‐related gene expression was enhanced with T. Specifically, *IGF‐1Ec* up‐regulation is purported to correlate with transcriptional activity^82^ and initiation of satellite cell proliferation,^83^ while *IGF1‐Ea* expression is correlated with increased translation^82^ and promotion of myogenic differentiation.^83^ Both intramuscular IGF‐1 and T independently stimulate AR and thereafter expression of other anabolic genes^72^, ^77^, ^78^; therefore, it is highly likely that an IGF‐1 signalling axis played a role in the efficacy of T therapy. Here, we also show augmented expression of myogenic regulatory factors (MRFs) and fast fibre‐type‐specific mRNA expression (i.e. *MHCIIa* expression^83^) in line with fast fibre, lower body, and whole‐body hypertrophy with T. This supports previous work^77^, ^84^ demonstrating a pro‐myogenic role for T; for example, T increased the transformation of pluripotent precursor cells down the myogenic lineage. It has been shown that single bouts of RE or short‐term RET are sufficient to increase the abundance of MRFs mRNA species in young participants.^73^, ^76^ However, in the present study, in line with past work in older age,^83^, ^85^ in the P group, these regulatory genes did not change either after an acute bout of RE or after 6 week RET with the exception of *C‐myc* (which controls cell growth and ribosomal biogenesis^20^). We speculate this contributed to blunted adaptations to RET in P^20^ while helping explain greater muscle mass accretion with T therapy.

In addition to declines in muscle mass and function with age, muscle mitochondria are also subject to age‐related remodelling. For instance, it was reported that ageing is associated with decreases in PGC1‐α mRNA (a main regulator of mitochondrial biogenesis and oxidative capacity^86^), OxPhos capacity, mitochondria enzyme activity (e.g. CS), mtDNA content, and increases in oxidative stress, which all result in an impaired mitochondrial function^87^. Crucially, exercise, including RET, is known to induce mitochondrial adaptations. For instance, 12 week RET augmented mitochondrial content,^88^ volume,^89^ and respiratory capacity,^90^ in younger participants. Further, 6 month RET increased mitochondrial mass in older women^91^ and mitochondrial function/transcriptome activity in older men.^92^ As we showed in our P study group, short‐term RET did not augment mitochondrial CS activity in older men; but similar to short‐term (i.e. 7–9 weeks) endurance training adjuvant to T therapy in mice,^93^, ^94^ we showed RET coupled to T therapy augmented PGC1‐α/Tfam mRNA, mitochondrial CS activity,^2^ and protein levels of complex IV (cytochrome *c* oxidase subunit^85^) and V (ATP synthase subunit)^85^ (not mtDNA copy number). Possible mechanisms include heightened activation of IGF‐1/MAPK pathways^87^, ^95^ and augmented MRFs mRNA (e.g. *Myogenin*)^96^ during RET, as observed in the T study group, which consequently mediate mitochondrial gene expression.^87^, ^96^ Furthermore, because mitochondrial gene expression (i.e. PGC1‐α) is regulated by Akt/mTOR signalling,^97^, ^98^ which were all up‐regulated with T, this may also explain our observations of greater CS activity with adjuvant T therapy. Moreover, sustained muscle growth and MPS during RET in the T group likely required greater increases in mitochondrial function or volume due to the increased energy demands of intracellular protein accretion.^89^ Previous work has also shown that the AR^98^ and systemic levels of T were positively associated with cytochrome *c* oxidase,^98^, ^99^ CS^100^ activity, and mitochondrial protein synthesis, in addition to Tfam and PGC1‐α expression,^98^ culminating in enhanced mitochondrial biogenesis and respiratory capacity and function.^99^ Conversely, AR and T deficiency has been associated with lower levels of PGC1‐α expression^101^ resulting in decreases in OxPhos capacity.^102^ In summary, greater MPS, myofibrillar protein accretion, and consequent muscle hypertrophy alongside greater mitochondrial adaptations^2^ in the T therapy group demonstrate the efficacy of adjuvant T in yielding positive hypertrophic and energetic adaptations.

We conclude that administration of T coupled to RET is an effective short‐term (6 weeks) intervention to overcome age‐related deficits in the responsiveness of older muscle to RET. Short‐term RET adjuvant to T was also well tolerated and could be useful as both pre‐habilitation and rehabilitation interventions in elective surgery and other clinical procedures in older populations. Nevertheless, it is important to be cognizant of potential adverse effects. T therapy has been associated by (non‐significant) increases in some disorders, for example, cardiovascular risk^103^ and/or prostate hypertrophy.^104^ While randomized controlled trials have been insufficiently powered to detect differences in the rates of adverse events,^105^ some have reported an increased risk of stroke and/or myocardial infarction,^106^ whereas others have reported to be uncertain of such effects.^105^, ^107^, ^108^, ^109^ No adverse events were noted during or after completion of the present study in older men and a major benefit of our approach is the short‐term efficacy of therapy. Finally, we reveal the likely mechanisms underlying the effect of T therapy in relation to ‘overcoming' aspects of age‐related anabolic resistance, vis‐à‐vis, elevating protein turnover (with greater increases in MPS), enhancing translational efficiency and capacity, and inducing pro‐myogenic and T handling gene regulation. Given links between declining T and muscle ageing in women,^110^ similar studies in women are likely to deliver similar benefits. Short‐term T administration may have a role in the treatment of frailty in older men without inducing any adverse side effects; however, older men receiving T therapy should be carefully monitored because of its potential risks.

## Conflict of interest

None declared.

## Funding

This work was supported by the Medical Research Council (grant numbers MR/R502364/1 and MR/P021220/1) as part of the MRC‐ARUK Centre for Musculoskeletal Ageing Research awarded to the Universities of Nottingham and Birmingham, and the National Institute for Health Research, Nottingham Biomedical Research Centre.
